# Skin resistance to water gain and loss has changed in cane toads (*Rhinella marina*) during their Australian invasion

**DOI:** 10.1002/ece3.6895

**Published:** 2020-10-11

**Authors:** Georgia K. Kosmala, Gregory P. Brown, Richard Shine, Keith Christian

**Affiliations:** ^1^ School of Life and Environmental Sciences University of Sydney Sydney NSW Australia; ^2^ Department of Biological Sciences Macquarie University Sydney NSW Australia; ^3^ Research Institute for the Environment and Livelihoods Charles Darwin University Darwin NT Australia

**Keywords:** abiotic challenges, Anura, *Bufo marinus*, physiology, water balance

## Abstract

The water‐permeable skin of amphibians renders them highly sensitive to climatic conditions, and interspecific correlations between environmental moisture levels and rates of water exchange across the skin suggest that natural selection adapts hydroregulatory mechanisms to local challenges. How quickly can such mechanisms shift when a species encounters novel moisture regimes? Cutaneous resistance to water loss and gain in wild‐caught cane toads (*Rhinella marina*) from Brazil, USA (Hawai'i) and Australia exhibited strong geographic variation. Cutaneous resistance was low in native‐range (Brazilian) toads and in Hawai'ian populations (where toads were introduced in 1932) but significantly higher in toads from eastern Australia (where toads were introduced in 1935). Toads from recently invaded areas in western Australia exhibited cutaneous resistance to water loss similar to the native‐range populations, possibly because toads are restricted to moist sites within this highly arid landscape. Rates of rehydration exhibited significant but less extreme geographic variation, being higher in the native range than in invaded regions. Thus, in less than a century, cane toads invading areas that impose different climatic challenges have diverged in the capacity for hydroregulation.

## Graphical abstract

The invasion of cane toads from South America to Hawai'i and Australia has been accompanied by substantial shifts in the ability of the skin to restrict rates of water flow. Over a timeframe of less than a century, the toad invasion has generated major changes in a fundamental aspect of rates of abiotic exchange between the organism and its environment.

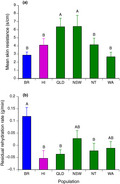

## 1 INTRODUCTION

The integument of most amphibians is highly permeable to water, restricting the activity of these animals to times and places where water is readily available or can be rapidly replenished (Buckley & Jetz, 2007; Lofts, 2012; Vitt & Caldwell, 2013; Wygoda, 1984). Although an amphibian's behavior, physiology, and morphology all affect the rate at which it exchanges water with the environment (Lofts, 2012; Vitt & Caldwell, 2013), the skin's permeability to water ultimately limits the rate at which water can be gained or lost (Hillyard et al., 1998; McClanahan & Baldwin, 1969; Spotila & Berman, 1976; Tracy, 1975). Some amphibians exhibit “waterproofing” adaptations (such as the skin secretions of *Litoria caerulea* and *Polypedates maculatus*, and cocoons of *Cyclorana australis*) that allow them to survive in arid conditions (Christian & Parry, 1997; Lillywhite et al., 1997), but most amphibians instead exhibit high rates of water flux between the body and the external environment (Lofts, 2012; Spotila & Berman, 1976).

**FIGURE 1:**
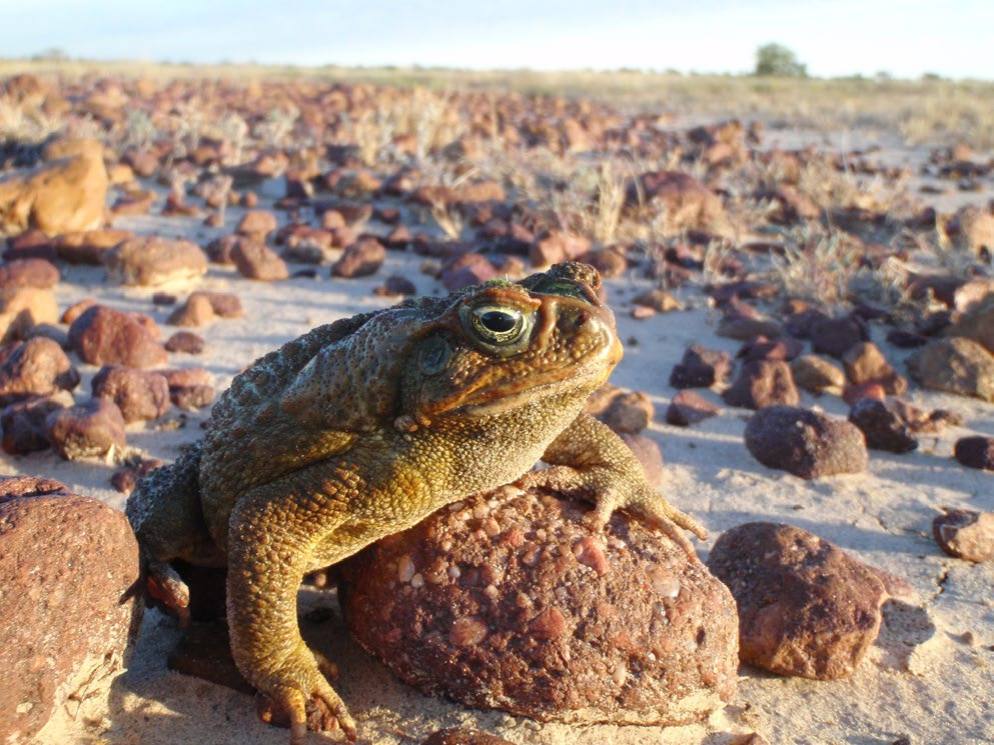
Adult cane toad, *Rhinella marina*, in arid habitat near Longreach, Queensland. Photograph by R. Shine

Interspecific correlations between skin permeability and environmental aridity suggest that a species' geographic distribution is constrained by its ability to maintain positive water balance (greater influx of water than efflux) under the conditions it encounters (Seebacher & Franklin, 2011; Tingley et al., 2012). If so, we would predict a biological invasion from one hydric regime to another to impose selection on rates of desiccation and rehydration, and hence on skin permeability. The cane toad (*Rhinella marina*, formerly *Bufo marinus*; see Figure 1) provides an ideal model system with which to test that prediction. Native to relatively aseasonal, well‐watered landscapes of South America, the species was translocated to much drier habitats on the Hawai'ian islands (Easteal, 1981; Ward‐Fear et al., 2016) and then Australia (Easteal et al., 1985; Freeland & Martin, 1985). Previous research has documented shifts in the species' morphology, physiology, and behavior coincident with translocation (e.g., Gruber et al., 2017; Hudson et al., 2016, 2017; McCann et al., 2014). We therefore quantified rates of evaporative water loss and gain in cane toads from populations within their native range (Brazil), their translocated range in Hawai'i (USA), and their current range in Australia to explore if skin permeability to water also has changed during translocation and range expansion. Specifically, we expected that colonization of seasonally arid environments in Australia would favor a shift toward higher cutaneous resistance to water flow.

## 2 MATERIALS AND METHODS

### 2.1 Study species

Cane toads are large “true toads” (family Bufonidae) native to an extensive area of South America (Bessa‐Silva et al., 2020; Zug & Zug, 1979), occurring in a variety of habitats including grassland, woodland, sand dunes, rainforest, mangroves, and anthropogenically disturbed areas. Whereas the dorsal skin is tough and leather‐like, the smoother ventral skin (especially the highly permeable pelvic patch) is responsible for most of the organism's water uptake (Parsons et al., 1993). The species' Latin name *Rhinella marina*, and one of its common names—the marine toad—come from its putative resistance to higher levels of salinity than are tolerated by most other anurans (Liggins & Grigg, 1985; Uchiyama & Konno, 2006; Wijethunga et al., 2016).

These terrestrial anurans were imported from French Guiana to Puerto Rico to control insect pests in sugar‐cane plantations; from there, 150 toads were translocated to Hawai'i in 1932, and 101 descendants of the Hawai'ian toads were introduced to northeastern Australia in 1935 (Freeland & Martin, 1985; Lever, 2001; Shine, 2018). Both in Hawai'i and Australia, the toads now occupy xeric as well as mesic habitats. In Hawai'i, the toads were released in plantations on leeward as well as windward coasts, but are currently restricted to anthropogenically moistened areas (such as golf courses) in the drier regions (Ward‐Fear et al., 2016). In Australia, toads were released in mesic coastal Queensland (QLD), but have since spread into seasonally arid habitats of Western Australia (WA) and the Northern Territory (NT), as well as colder montane habitats in New South Wales (NSW: for details see Feit et al., 2015; McCann et al., 2014; Newell, 2011; Shine, 2010; Tingley et al., 2012). Cane toads thus occupy a wider range of climatic conditions in Australia than in their native range (Tingley et al., 2014).

### 2.2 Sampling locations

We collected adult toads (both males and females, ranging from 43 to 313 g; mean = 109.9 g ± 33.5 g *SD*) from locations in their native range (Brazil), in Hawai'i (USA), and in Australia (see Table 1). All toads were collected by hand at night, placed in damp cloth bags, and kept in a moist, cool environment. Following capture, toads were transported to local laboratory facilities for the experiments.

**Table 1:** Collection sites of cane toads used for the current study, with data on year of cane toad introduction and annual rainfall

Country	State	Location	Year of introduction	Mean annual rainfall (monthly range)	Mean temperature (monthly range)
Brazil	PA	Alter do Chão	Native range	1,991 mm (34–346)	25.9°C (25.1–26.9°C)
	AM	Manaus	Native range	2,145 mm (56–295)	27.4°C (26.9–28.2°C)
USA	HI	Hilo	1932	3,459 mm (177–397)	23.1°C (21.7–24.6°C)
		Kailua‐Kona	1932	862 mm (55–88)	23.5°C (22.0–24.9°C)
Australia	WA	Kununurra	2011	720 mm (0–186)	28.8°C (23.3–32.6°C)
		Oombulgurri	2013	718 mm (0–181)	29.4°C (24.3–32.9°C)
	NT	Leaning Tree Lagoon	2006	1,500 mm (1–364)	27.2°C (23.9–29.4°C)
		Katherine	2010	1,009 mm (0–250)	27.5°C (22.1–31.6°C)
	QLD	Charters Towers	1953	692 mm (8–142)	23.2°C (17.3–27.4°C)
		Townsville	1935	1,111 mm (9–275)	24.1°C (19.0–27.6°C)
	NSW	Brooms Head	2005	1,471 mm (49–188)	19.2°C (13.8–23.6°C)
		Tabbimoble	2010	1,558 mm (52–193)	19.4°C (14.0–23.6°C)

Note

PA = Alter do Chão, Pará, AM = Manaus, Amazonas, HI = Hawai'i, WA = Western Australia, NT = Northern Territory, QLD = Queensland, NSW = New South Wales. Dates of toad introduction from Lever (2001) and Urban et al. (2007). Climatic data (from climate‐data.org) show mean annual values, and range of mean monthly values for each site.

**FIGURE 2:**
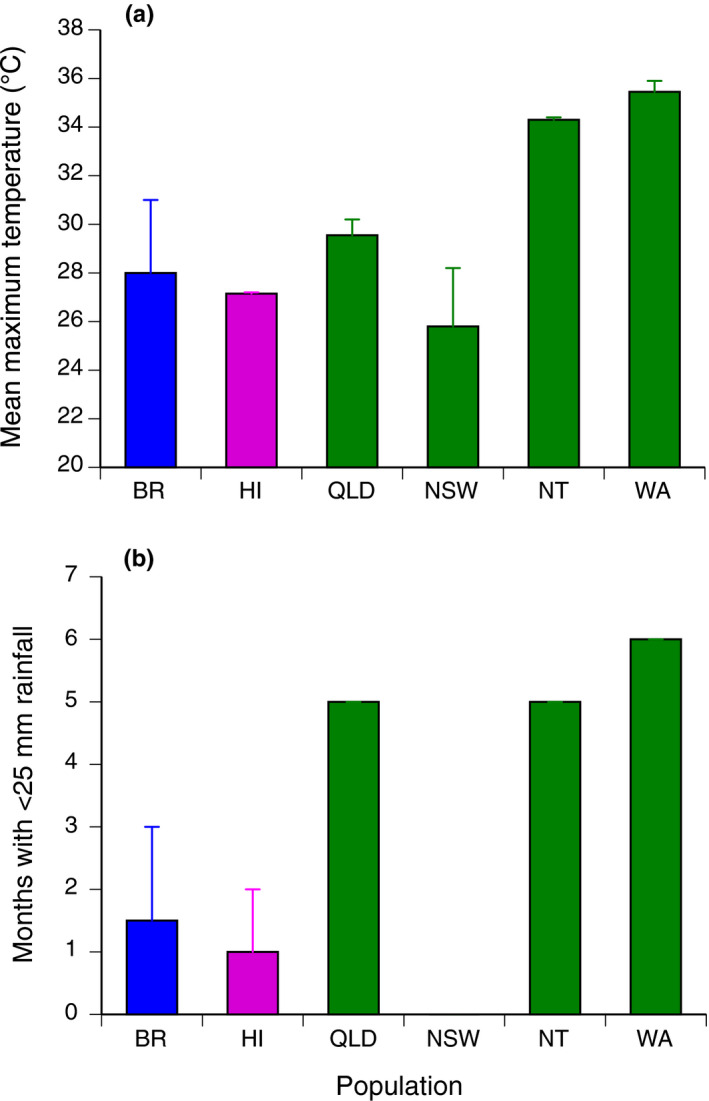
Climatic conditions of the collection sites. (a) Mean maximum temperature, and (b) number of months with less than 25 mm of rainfall (locations with higher bars have more months of very low rainfall, showing dry conditions). BR = Brazil, HI = Hawai'i, QLD = Queensland, NSW = New South Wales, NT = Northern Territory, WA = Western Australia

Toads from the native range were collected in Manaus, Amazonas (AM) and Alter do Chão, Pará (PA) in Brazil during January and February 2015, a warm and wet time of year (Table 1). We collected toads on the island of Hawai'i (HI, United States of America) during June and July 2015, from sites in the extreme east (Hilo) and extreme west (Kailua‐Kona) of the island. The windward eastern side of the island is humid and warm (Hawai'i wet; HW), whereas the leeward western side is much drier (Hawai'i dry; HD) due to a rain shadow effect coupled with highly porous volcanic soils (Ward‐Fear et al., 2016) (Table 1, Figure 2). However, the toads inhabit moist areas in both of these regions because of anthropogenically provided water (Ward‐Fear et al., 2016). In Australia, we collected toads from eight sites. Two sites were in Western Australia (Oombulgurri sampled in November 2014, Kununurra in October 2015), in the extreme west of the species' range close to the invasion front (<2 years post‐colonization) where the climate is hot (annual average close to 30°C) year‐round, and seasonally arid. Another two sites were in the Northern Territory (Katherine, Leaning Tree Lagoon, both in August 2015), where the climate is less harsh (longer wet season) than the Western Australian sites. One Queensland site (Townsville in September 2015) experiences cooler but seasonally arid conditions, whereas the other (Charters Towers, also in September 2015) is drier for much of the year and very hot in summer. Lastly, the two sites in New South Wales (Brooms Head and Tabbimoble, both in October 2015) are close to the current southeastern invasion front, where the climate is cooler and generally moist (see Table 1, Figure 2 and Kosmala et al., 2017 for details of site locations, invasion history, climatic conditions, and sample sizes). Climate data were sourced from Climate‐Data.org.

### 2.3 Husbandry

After capture, we allowed the toads to acclimate in laboratory conditions for 2 weeks, fed them crickets and mealworms, and provided ad libitum access to water and shelter. The room was set to 25°C, with a 12:12 hr light cycle (for details of methods see Kosmala et al., 2017). All animals were housed and tested in their respective countries of collection, in near‐identical conditions. Prior to measurements, we emptied the toads' bladders by gently applying pressure to the abdomen until urine was released.

### 2.4 Rates of evaporative water loss and cutaneous resistance

We used a closed system with a positive airflow, with silica gel cylinders at the intake and humidity probes at the outflow of the system (a similar system is described in detail in Young et al., 2005). Dry airflow (<1% relative humidity) was adjusted to 1 L/min, and we recorded baseline humidity values for the system for at least 20 min (after humidity values stabilized) prior to the toads being introduced to the system (average relative humidity 0.048% ± 0.064). Animals were held within an airtight cylindrical container of 1‐L capacity. After a toad was placed into the container, we waited for it to adopt a water‐conserving posture (WCP; with limbs folded beneath the body: see Withers et al., 1984), then recorded temperature and humidity of air that had passed across the toad's body for at least 20 min (as long as the toad remained in WCP). We then removed the animal from the container, and we continued to record temperature and humidity within the airflow chamber for another 20 min to ensure that baseline values had not changed. After it was removed from the airflow system, the toad was weighed and placed in a water tub in its home cage to rehydrate. We also measured rates of evaporative water loss of agar models (3% concentration) to measure the rate of evaporation from a free water surface (i.e., without cutaneous resistance), so that we could calculate individual cutaneous resistance to water loss (method validated by Spotila & Berman, 1976 and Christian et al., 2017). The agar models were made using molds taken from cane toads in WCP and were placed in the chambers such that the ventral portion was hidden (as is the case for a toad in WCP). Molds were made from 21 cane toads collected from the Darwin region, ranging in size from 9.5 to 365 g, and the water loss from the corresponding agar models was used to calculate total resistance, which corresponds to the boundary layer resistance of a toad of the same size. A regression equation was calculated for the relationship between toad mass and the total resistance of the agar models (resistance = 0.7222 + 0.0104 X mass; *F*_1,19_ = 46.8, *p* < .001; *r* ^2^ = 71.1%), and this relationship was used to calculate the boundary layer resistance for each toad. The variability around the regression line was due, in part, to variability in the relationship between mass and surface area, resulting from differences in body condition. The mean boundary layer resistance was 1.9 s/cm (*SD* = 0.35), and the range was 1.4 (from the smallest toad) to 4.0 s/cm (from the largest toad).

### 2.5 Rates of rehydration

Following our measures of water flow, these animals were then used in studies to assess effects of desiccation on locomotor performance (Kosmala et al., 2017), which included experimental dehydration in dry air. Prior to all experiments, toads were kept in water for 2 hr to ensure full hydration. The toads were then weighed (after emptying the bladder: see above), placed in desiccating conditions, and reweighed frequently until they reached the desired reduction in mass. Once it reached 70% of its initial body mass, the toad was weighed, placed in a container with ~0.5 mm depth of water, and reweighed every 2 min (after pat‐drying with paper towel) for 14 min to measure rates of rehydration (McClanahan & Baldwin, 1969).

### 2.6 Statistical analyses

Sites were grouped to form the populations as follows: BR = Alter do Chão and Manaus (Brazil), HI = Hilo and Kailua‐Kona (Hawai'i), WA = Kununurra and Oombulgurri (Western Australia), NT = Katherine and Leaning Tree Lagoon (Northern Territory), QLD = Charters Towers and Townsville (Queensland), and NSW = Brooms Head and Tabbimoble (New South Wales). In addition to examining effects of location, we tested if sex or body mass had an influence on either cutaneous resistance or rehydration. If either sex or body mass had an effect, we used ANCOVA with the additional parameter in question as a covariate; otherwise we used ANOVA.

Cutaneous resistance was not related to toad body mass (*r* ^2^ = 0.05, *p *= .19), and we therefore performed a simple ANOVA with location (country/state) as the factor. Rehydration rate was however significantly affected by a toad's mass (see below). Therefore, we used ANCOVA with mass as a covariate to assess differences in rehydration rate among locations (country/state).

Post hoc tests (Tukey's test) were performed to locate significant differences among groups indicated by ANOVA and ANCOVA. Values of rehydration rate and body mass were ln‐transformed prior to analyses to meet assumptions of normality, homoscedasticity, and homogeneity of variance. Data on cutaneous resistance, rehydration rate, and body mass were not strongly co‐linear (all Pearson's *r* < 0.31), and residuals from analyses did not violate assumptions of homoscedasticity. Analyses were performed using JMP 11 (SAS Institute, Cary, NC), using an alpha level of *p < *.05.

## 3 RESULTS

Preliminary analyses using site of collection as a factor in ANOVA revealed no significant differences in cutaneous resistance among sites within each broader location (country or state), and thus we combined sites within these broader locations for further analysis. Likewise, cutaneous resistance and rehydration rate did not differ between sexes (both *F* < 1.55, both *p *> .22) and thus sex was excluded from further analyses.

There was a weak but statistically significant negative correlation between cutaneous resistance and rehydration rate (Spearman *r *= −0.19, *p* = .03).

### 3.1 Geographic variation in cutaneous resistance

**FIGURE 3:**
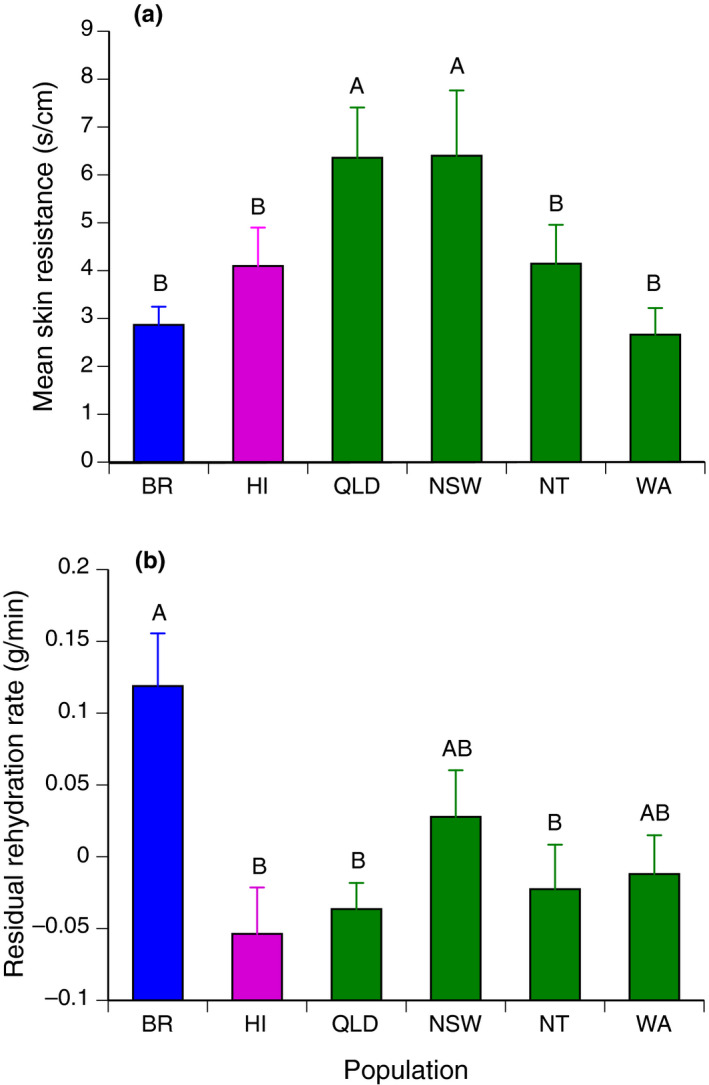
Values of (a) cutaneous skin resistance (s/cm) (±*SE*), and (b) residual rehydration rates (g/min) (±*SE*) of cane toads collected in native and invasive populations. BR = Brazil, HI = Hawai'i, QLD = Queensland, NSW = New South Wales, NT = Northern Territory, WA = Western Australia. Letters indicate groups obtained by post hoc (Tukey) test; different letters indicate significant differences. Residual rehydration rate values are adjusted for toad body mass

ANOVA with collecting location (country or state) as the factor showed significant geographic variation in cutaneous resistance (*F*_5,158_ = 18.94, *p *< .001; Tukey post hoc tests identified two groups, with NSW and QLD similar to each other, and both significantly higher than the other four regions). Thus, cutaneous resistance to water loss was relatively low in cane toads from the native range and Hawai'i, high in relatively mesic areas of eastern Australia (NSW and QLD), but low in populations from seasonally arid sites within western Australia (NT and WA) (Table 2, Figure 3a).

**Table 2:** Sample sizes, body masses, rates of water loss, skin resistance, and rehydration rates of cane toads used in experimental protocols of the current study (mean ± *SD* for applicable variables)

Country	State	Location	Number of toads	Mass (g)	SVL (mm)	Collection date	Mean temperature^a^ (°C)	Mean rainfall^a^ (mm)	Rate of water loss (mg/min)	Skin resistance (s/cm)	Rehydration rate (g.mins^−1^)
Brazil	PA	Alter do Chão	17	110.2 ± 23.6	109.1 ± 7.3	Feb 2015	26.1	7.7	7.2 ± 1.4	2.9 ± 2.1	0.6 ± 0.2
	AM	Manaus	13	76.6 ± 24.2	97.6 ± 2.7	Jan 2015	25.6	3.8	5.9 ± 0.8	2.8 ± 2.1	0.5 ± 0.2
USA	HI	Hilo	11	104.1 ± 13.7	109.1 ± 4.6	Jun 2015	25.2	1.4	10.0 ± 1.5	3.3 ± 1.0	0.4 ± 0.2
		Kailua‐Kona	15	125.8 ± 31.4	110.4 ± 8.2	Jun 2015	26.8	2.0	10.4 ± 1.5	4.7 ± 2.5	0.3 ± 0.1
Australia	WA	Kununurra	17	90.6 ± 21.8	105.3 ± 5.9	Oct 2015	30.1	0.4	10.2 ± 2.0	2.8 ± 1.0	0.4 ± 0.1
		Oombulgurri	6	104.8 ± 13.1	122.1 ± 5.6	Nov 2014	32.9	3.9	10.9 ± 1.4	2.4 ± 2.1	0.5 ± 0.1
	NT	Leaning Tree Lagoon	10	143.7 ± 24.0	118.0 ± 4.1	Aug 2015	23.6	0.0	10.7 ± 1.4	4.5 ± 1.6	0.5 ± 0.2
		Katherine	16	130.9 ± 58.4	112.3 ± 11.8	Aug 2015	23.3	0.0	9.9 ± 2.0	3.9 ± 2.1	0.4 ± 0.1
	QLD	Charters Towers	20	114.1 ± 31.1	110.1 ± 19.2	Sept 2015	25.9	0.1	8.8 ± 1.7	6.3 ± 2.1	0.4 ± 0.1
		Townsville	17	92.3 ± 16.4	103.8 ± 6.7	Sept 2015	23.1	0.0	8.1 ± 1.6	6.4 ± 1.7	0.4 ± 0.1
	NSW	Brooms Head	10	120.1 ± 23.4	108.6 ± 8.4	Oct 2015	20.5	0.7	9.7 ± 2.0	6.5 ± 2.6	0.4 ± 0.1
		Tabbimoble	12	113.1 ± 29.3	106.2 ± 6.5	Oct 2015	20.0	1.2	8.9 ± 1.6	6.3 ± 1.7	0.5 ± 0.1

PA = Alter do Chão, Pará, AM = Manaus, Amazonas, HI = Hawai'i, WA = Western Australia, NT = Northern Territory, QLD = Queensland, NSW = New South Wales.

^a^ During month of collection.

There was a significant negative correlation between average cutaneous resistance at each site and the mean temperature recorded at the site (Spearman *r* = −0.78, *p* = .003), but no significant correlation between average cutaneous resistance and average rainfall (Spearman *r* = −0.05, *p* = .88).

### 3.2 Geographic variation in rates of rehydration

ANCOVA indicated that rehydration rates were positively related to body mass (*F*_1,124_ = 17.65, *p *< .0001) and also significantly related to population of origin (*F*_5,124_ = 3.70, *p *< .004). The interaction between mass and location was not significant (*F*_5,124_ = 0.37, *p *= .87). An ANOVA on the residuals of the regression of rehydration rates on body mass indicated that rehydration rates for Brazilian toads were higher than for all populations in either of the other two countries (Figure 3b).

There were no significant correlations between average rehydration rate at each site and the mean temperature or rainfall (both Spearman *r* < 0.42, *p* > .18).

## 4 DISCUSSION

The rate at which an anuran loses water from its body is a critical aspect of its biology, restricting the times and places where it can be active (Hillyard et al., 1998; McCann et al., 2014; Seebacher & Franklin, 2011; Toledo & Jared, 1993; Young et al., 2005). We found differences in aspects of hydric biology across populations of cane toads. Toads from two regions in eastern Australia (QLD and NSW) exhibited cutaneous resistances significantly higher than seen in the native range of the species (Brazil), or in the “stepping stone” populations in Hawai'i (USA) from which the eastern‐Australian populations were founded in 1935. Surprisingly, however, northwestern‐Australian populations (the most recently invaded sites: NT and WA) had cutaneous resistance levels similar to those of conspecifics in the native range. Our results for Northern Territory toads were slightly higher than those of Young et al. (2005) (cutaneous resistance = 1.7 ± 0.7 s/cm).

What mechanisms underlie these shifts in hydroregulatory abilities of cane toads across northern Australia? First, the differences in cutaneous resistance might be the result of evolutionary changes that have occurred as the toads have adapted to the novel environments they have encountered during the westwards invasion of northern Australia. Another possibility is that, rather than genetic differences, the differences among populations are the result of developmentally plastic responses to local environments. A third possibility is that toads from eastern Australia may have exhibited an acute (temporary) physiological response that affected their cutaneous resistance during the period they were measured. Acute changes in water flux have been documented in toads in response to external agents (Dohm et al., 2001) and to pharmacological agents (biochemical blockers and stimulants) that affect cutaneous blood flow (Burggren & Vitalis, 2005; Hillyard et al., 1998). Acute changes to resistance can occur in frogs in response to environmental temperature (Buttemer & Thomas, 2003; Tracy et al., 2006). Because we relied on measurements from field‐caught animals, we cannot tease apart the degree to which geographic divergence was driven by evolved (heritable) changes versus developmentally plastic or acute responses to local environments. To distinguish among these possibilities, additional field sampling and studies on offspring raised under standardized conditions would be needed.

If the changes in resistance are an evolutionary response, then it could have occurred by one of two patterns: either the change to high cutaneous resistance occurred in Queensland but the trend reversed as the toads moved west over 80 years, or, alternatively, the westbound colonists retained the ancestral state (of low cutaneous resistance) while the toads from eastern Australia evolved high cutaneous resistance later, after the westwards spread of toads had already commenced. Two patterns suggest that the former scenario is more plausible. First, the toad invasion front expanded very slowly in the decades immediately post‐translocation (Freeland & Martin, 1985; Kearney et al., 2008; Phillips et al., 2007; Urban et al., 2007), consistent with the time needed for adaptation of traits (such as cutaneous resistance) to deal with novel climatic challenges (e.g., Aikio et al., 2010; Kowarik, 1995). Second, if high cutaneous resistance did not evolve in Queensland toads until after the westwards invasion had begun, we would have expected low cutaneous resistance in toads from the southern (New South Wales) front due to retention of the ancestral condition (the western and southern fronts expanded at about the same time: Urban et al., 2007). Instead, toads from southern (New South Wales) populations had high cutaneous resistance, like Queensland conspecifics.

In contrast to cutaneous resistance, rates of rehydration showed a simpler pattern. The rates at which toads regain water have decreased over the course of the toad invasion, with native‐range (Brazilian) animals taking up water more rapidly than did those from either Hawai'i or Australia. Thus, mesic conditions within the native range appear to have favored a system of both gaining and losing water rapidly. In contrast, more arid conditions in Hawai'i and Australia have resulted in toads exhibiting greater resistance to water loss in some invaded sites but not others; and a lower rate of rehydration relative to Brazilian toads.

The rate of hydration also depended on body mass, reflecting allometric effects. Relative to internal volume, larger toads have a relatively smaller surface area (across which they gain water) than do smaller conspecifics (McClanahan & Baldwin, 1969; Tracy, 1975; Withers et al., 1984).

Interspecific comparisons suggest that species of anurans exposed to desiccating conditions (e.g., arid or arboreal habitats) tend to exhibit higher cutaneous resistance to water loss (Spotila & Berman, 1976; Tingley et al., 2012; Titon & Gomes, 2017; Wygoda, 1984; Young et al., 2005). This pattern is consistent with the ancestral condition of low cutaneous resistance to evaporative water loss seen in toads from Brazil and the wetter site in Hawai'i. Although the low resistance to water loss of toads from dry sites in Hawai'i (HD) does not fit well with that trend, toads in these sites actually live in anthropogenically moistened habitats where water is freely available (Ward‐Fear et al., 2016). Similarly, although many sites within the range of toads in the Northern Territory and Western Australia are semi‐arid, the toads survive the dry season by inhabiting areas with reliable moisture (Feit et al., 2015; Tingley & Shine, 2011; Tracy et al., 2014). Counter‐intuitively, then, toads in very arid landscapes may live in microhabitats where water is freely available (Tingley & Shine, 2011).

When toads were translocated from Hawai'i to Queensland in 1935, they encountered a climate where precipitation is hotter and more seasonal than in the native range (Table 1; Tingley et al., 2014). However, the toads' subsequent invasion into seasonally arid habitats in the western part of Australia was not accompanied by an increase in cutaneous resistance. In addition to inhabiting wetter sites within this arid location (Feit et al., 2015; Tingley & Shine, 2011; Tracy et al., 2014), a high rate of evaporative water loss may benefit toads in this region because of the extremely high ambient temperatures experienced through most of the year (Figure 2; and see Kosmala et al., 2017 and McCann et al., 2018 for climatic data). Among the 12 study sites, skin resistance was negatively correlated with average temperature. Thus, evaporative cooling (Borgnakke & Sonntag, 2016; Buttemer & Thomas, 2003; Tracy et al., 2006) might enable toads to deal with otherwise‐lethal heat loads (Kosmala et al., 2020). Analogously, Gila monsters increase cutaneous and cloacal evaporative water loss in hot weather (DeNardo et al., 2004). A feedback between body temperature and rates of evaporative water loss (cooler cane toads lose water less rapidly: Malvin & Wood, 1991) might reduce the hydric costs of evaporative cooling.

Our results add two additional variables—rates of water gain and cutaneous resistance to water loss—to the list of functional traits that exhibit geographic variation within cane toads, including across different sites within their invaded range within Australia (e.g., Hudson et al., 2016; Kosmala et al., 2017; McCann et al., 2018). These data demonstrate that anurans can adjust fundamental aspects of their interactions with environmental factors rapidly, if the taxon in question is exposed to abiotic challenges that differ strongly from those which it experiences in the native range.

## CONFLICT OF INTEREST

None declared.

## AUTHOR CONTRIBUTIONS

**Georgia Karoline Kosmala:** Conceptualization (equal); investigation (equal); methodology (equal); writing–original draft (equal). **Gregory P. Brown:** Data curation (equal); formal analysis (equal); investigation (equal); methodology (equal); project administration (equal). **Richard Shine:** Conceptualization (equal); formal analysis (equal); funding acquisition (equal); resources (equal); supervision (equal); writing–review and editing (equal). **Keith Christian:** Conceptualization (equal); data curation (equal); formal analysis (equal); investigation (equal); methodology (equal); supervision (equal).

## Data Availability

Data are available at Dryad https://doi.org/10.5061/dryad.ttdz08kwc.
